# Comprehensive Analysis of Photoreceptor Outer Segments: Flow Cytometry Characterization and Stress-Driven Impact on Retinal Pigment Epithelium Phagocytosis

**DOI:** 10.3390/ijms241612889

**Published:** 2023-08-17

**Authors:** Haoqian Liang, Qiang Wu, Xinzheng Victor Guo, Linda Chan, Tin Mao, Cinzia Stella, Axel Guilbaud, Julien Camperi

**Affiliations:** 1 Cell Therapy Engineering and Development, Genentech, South San Francisco, CA 94080, USA; liang.lainey@gene.com (H.L.); qiangw@gene.com (Q.W.); guox49@gene.com (X.V.G.); faith@gene.com (L.C.);; 2 Protein Analytical Chemistry, Genentech, South San Francisco, CA 94080, USA; stella.cinzia@gene.com

**Keywords:** retinal pigment epithelium cells, phagocytosis assay, photoreceptor outer segments, flow cytometry, fluorescence scanning

## Abstract

Phagocytosis is one of the key functions of retinal pigment epithelium (RPE) cells, which maintain photoreceptor health by removing photoreceptor outer segments (POSs) that are regularly shed. A deficiency in RPE function to phagocytose POSs may lead to vision loss in inherited retinal diseases and eventually to age-related macular degeneration (AMD) with geographic atrophy. Significant progress has been made in the field of cell replacement therapy for AMD using stem-cell-derived RPE. To test their function, RPE cells are incubated with purified bovine POSs for the demonstration of efficient binding, internalization, and digestion of POSs. Here, we present an image-based method to measure phagocytosis activity by using POSs labeled with a pH-sensitive fluorescent dye, which has low fluorescence at neutral pH outside of the cell and high fluorescence at low pH inside the phagosome. Further, we introduce a unique flow-cytometry-based method for the characterization of POSs by measuring specific markers for POSs such as rhodopsin and opsin. Using this method, we demonstrated a comparable quality of several bovine POS isolation batches and a reliable assessment of POS quality on RPE phagocytosis assay performance when subjected to different stress conditions. This work provides new tools to characterize POSs and insight into RPE phagocytosis assay development for the functional evaluation of RPE cells in the field of cell replacement therapy.

## 1. Introduction

Age-related macular degeneration (AMD) affects 200 million people worldwide and is a condition that causes damage to the central region of the retina, leading to irreversible vision loss and blindness [1,2]. The pathophysiology of AMD is complex and involves various cellular and molecular processes, during which retinal pigment epithelium (RPE) functions are impaired [3]. RPE cells play a crucial role in maintaining the health and function of the retina [4], and one critical process for maintaining retinal homeostasis is phagocytosis of the photoreceptor outer segments (POSs), which are shed daily and replaced by new segments produced to maintain visual function [5,6]. This phagocytosis activity involves the recognition and ingestion of POSs by RPE cells, followed by the degradation and recycling of the ingested material [7].

To develop effective drugs and therapies for AMD and related diseases, reliable methods for assessing retinal function and health are required. Over the last decade, significant progress has been made in the field of cell replacement therapy for AMD [8,9]. Several studies have investigated different methods for the isolation and expansion of RPE cells, including primary culture, tissue engineering approaches, and stem cell differentiation [10,11,12]. Recently, the transplantation of RPE cells derived from human embryonic stem cells (hESCs) or human induced pluripotent stem cells (hiPSCs) has shown promising results for functional visual recovery in animal models of retinal disease [13] and in patients with AMD in clinical trials [14,15].

RPE cells derived from hESCs and hiPSCs have been shown to exhibit functional and morphological properties similar to those of native RPE cells, including the expression of RPE-specific markers, secretion of retinal factors (e.g., PEDF, cytokines, interleukins), and the phagocytosis of POSs [16,17,18]. However, their ability to perform phagocytosis, a crucial function of native RPE cells, has not been fully characterized. Only a few studies have demonstrated the phagocytic ability of RPE cells derived from stem cells by using immunohistochemical staining [15], electron microscopy [19], and flow cytometry [20]. One important factor that can affect the reliability of these assays is the purity of the POS material used. Contaminants in the sample, such as debris and non-POS particles, can interfere with the analysis of phagocytosis and lead to erroneous results. Therefore, it is crucial to ensure the purity of the POS samples prior to conducting RPE phagocytosis assays.

Various analytical methods have been used for the characterization of POSs, including electron microscopy [21,22] and immunostaining [21]. Corley et al. also reported the use of a flow-cytometry-based method to assess the membrane heterogeneity of POS discs [23], but there have been no reported studies on the characterization of the POS material itself via flow cytometry. Flow cytometry is a powerful analytical tool for the analysis of cellular and subcellular particles, and has been widely used in biomedical research, especially in the study of immunology and cancer [24]. It has the advantages of high throughput, speed, and accurate quantification and characterization of cells or particles according to their shape, size, cellular content, and specific markers, making it an ideal tool to obtain valuable information on the POS material. In addition, this technology can help to exclude debris and non-POS particles through markers, ensuring the accuracy and reliability of the analysis.

In this paper, we present a novel flow-cytometry-based method for the precise characterization of POSs, using distinct markers like rhodopsin and opsin. These markers enable the differentiation of rod and cone outer segments, facilitating the separation of these two POS subtypes from potential impurities such as cellular debris or non-POS particles. Moreover, we extended our investigation by assessing the phagocytosis activity using an innovative-imaging-based approach. By subjecting POSs to various stress conditions, including heat and light stress, we gained insights into their potential impacts on the RPE phagocytosis potency assay. This two-fold approach not only contributes to the development of a reliable and accurate method for POS characterization, but also provides new insights into retinal phagocytosis and its complex pathology.

## 2. Results

### 2.1. Phagocytosis Assay

To demonstrate the phagocytosis ability of RPE cells, several in vitro phagocytosis assays can be performed, including flow-cytometry-based assays and conventional phagocytosis assays [25]. These studies have shown the engulfment, degradation, and recycling of shed photoreceptor outer segment (POS) tips via phagocytosis under different pH environments. Therefore, phagocytosis of POSs by RPE cells can be also measured using pH-sensitive fluorescent dyes that fluoresce upon ingestion into phagosomes, changing from a non-fluorescent state at neutral pH outside the phagocyte to a highly fluorescent state in the low pH environment of the phagolysosome (Supplementary Figure S1). The use of pH-sensitive fluorescent dyes to label POS particles allows rapid and efficient measurement of the phagocytic capacity of RPE cells using an imaging-based method.

**Figure 1:**
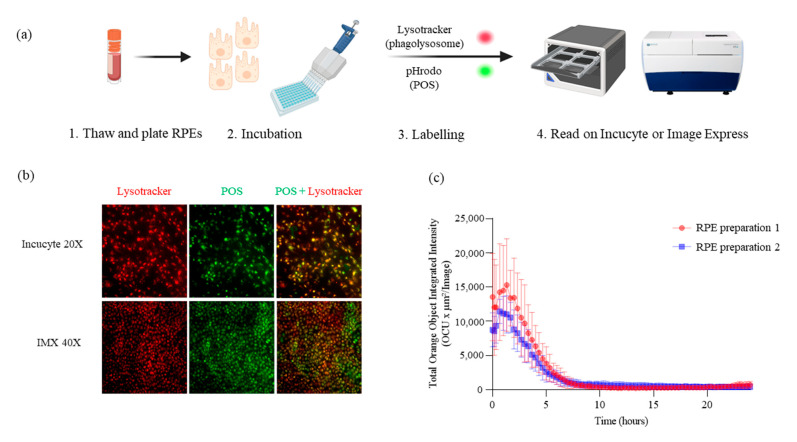
Assessing RPE phagocytosis capacity using an image-based methodology for a real-time quantitative live-cell imaging measurement. (**a**) Assay setup of the phagocytosis assay. RPEs were thawed, plated, and incubated. This step was followed by POS labeling with pH-sensitive conjugated probes (pHrodo dyes), and phagocytosis activity was then recorded on Incucyte^®^ or IMX *(images are adapted from Biorender.com*). Lysosomes and POSs were labeled with a LysoTracker red fluorescent dye ((**b**), left panels) or a pHrodo green dye ((**b**), middle panels) separately, or colocalized ((**b**), right panels). (**c**) To monitor the POS internalization by RPE, the total integrated intensity was collected for 24 h from two cell preparations. The difference between RPE preparation 1 and RPE preparation 2 is that preparation 2 was passed 2 more times. The phagocytosis experiments were performed in triplicate (*n* = 3), and the corresponding error bars based on standard deviation (SD) were reported.

Figure 1a presents a phagocytosis assay that combines two fluorescent dyes with an Incucyte^®^ or ImageXpress^®^ Micro Confocal system (IMX) for real-time quantitative live-cell imaging and analysis measurement. For this assay, lysosomes and POS particles were first labeled with a red fluorescent dye, LysoTracker, and a pHrodo green dye, respectively (Figure 1b, left panel). When RPE cells were added to POSs, the results show an overlap between green and red-orange objects in both human RPE (huRPE) and RPE samples (Figure 1b, right panel), indicating the colocalization of POSs and LysoTracker in RPE cells (Figure 1b). Therefore, the data support that POS particles are phagocytosed by functional RPE cells.

Assessing RPE phagocytosis activity over time is also important to understand the dynamic nature of the process. Quantitative analysis of RPE phagocytosis in the two different RPE preparations (*n* = 3 for each) presented in Figure 1c revealed the same transient increase in activity (216,272.9 ± 59,489.7 and 238,529.9 ± 24,483.0 OCU × um^2^/image) shortly after the initiation of the phagocytosis assay (1–2 h), followed by a gradual decline as the process continued and phagosomes still processed and degraded POSs within the RPE cells.

### 2.2. Characterization of POS Purity Using a Flow-Cytometry-Based Method Targeting Specific ROSs and COS Markers

**Figure 2:**
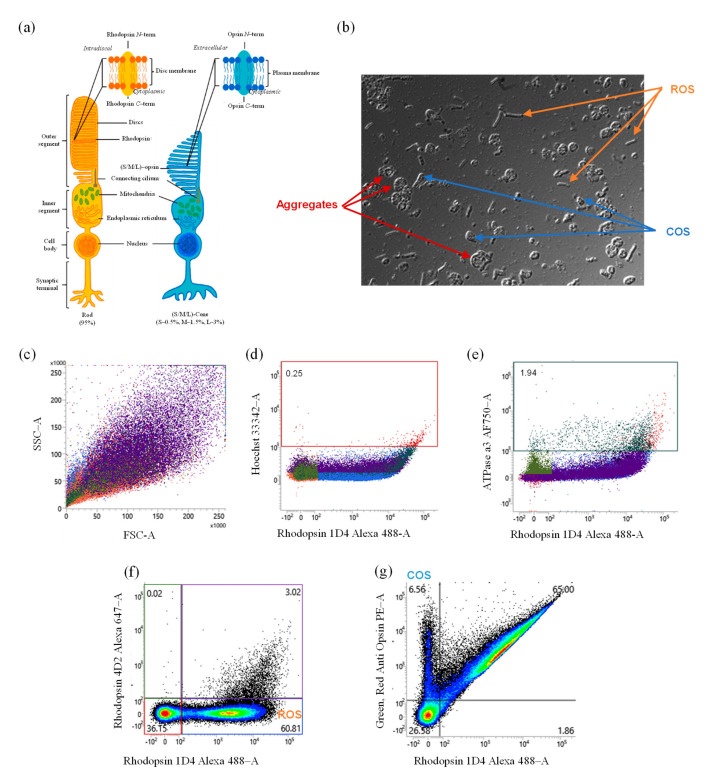
Characterization of POS population using microscopy and flow-cytometry-based methods. (**a**) Cellular structure representation of rods and cones, two cell subtypes of POSs. (**b**) Microscopy examination revealing ROS-shaped structure (orange arrows), COS-shaped structure (blue arrows), and POS aggregates (red arrows). (**c**) Flow-cytometry-based method isolation of POS population based on SSC−A (side scatter area, indicating the granularity) vs. FSC−A (forward scatter area, indicating the size) parameters. (**d**) Hoechst 33342−A or (**e**) ATPase a3 AF750−A was used to determine nucleated cells or impurities such as retina cellular debris or inner segments. Specific rhodopsin antibodies conjugated with Alexa Fluor^®^ with emission wavelengths at 488 and 647 nm were used to isolate the ROS population (**f**), while the COS population was identified from the lower left (LL) gate using an anti-opsin antibody conjugated to a PE fluorophore emitting at 488 nm (**g**). The number of events recorded for the flow cytometry panels was 100,000. A different gate was used to determine the corresponding population. In-house retinal extraction experiments were performed to generate a positive control for Hoechst 33342−A and ATPase a3 (see Supplementary Figure S2). Data obtained from POS batch 1.

As described in Figure 2a, there are two main subtypes of photoreceptor cells: rods and cones. The proportion of cones and rods varies depending on the region of the retina; however, in general, it is believed that bovine retina contains a higher proportion of rods (95%) than cones (5%) [26]. Both cones and rods have a similar structure, consisting of an inner segment, a cell body, and an outer segment. The outer segment is the part of the cell that contains the photo-pigment molecules responsible for absorbing light and initiating the signaling cascade that leads to neural activity. The cone outer segments (COSs) are typically shorter and wider than the rod outer segments (ROSs). The microscopic examination of a representative sample of POSs is presented in Figure 2b, showing the presence of rod-shaped and cone-shaped structures and aggregates.

The identity and purity of POSs were then examined via flow cytometry, first using FSC−A and SSC−A parameters, revealing a highly heterogeneous population of cells with a wide range of size and granularities (Figure 2c). The scatter plot showed a broad distribution of events, indicating that the population is not easily distinguishable based on these parameters alone. Additional analysis using a negative control such as Hoechst dye (Figure 2d)—labeling the DNA in the nucleus of the cells—showed that there was no discernable nuclear staining, suggesting that the population may be composed primarily of non-nucleated cells or fragments. The same observation was made with an ATPase antibody, a second negative control, after staining the inner part of the POSs and other cells (e.g., bipolar cells) of the retina [27]. As shown in Figure 2e, few ATPase-positive events were detected, suggesting that POS samples were not contaminated by inner segments or other cell impurities. However, in Figure 2f, after gating using two rhodopsin-specific antibodies, 1D4 Alexa 488 for the cytoplasmic/extra-discal *C*-terminal and 4D2 Alexa 647 for the intra-discal *N*-terminal), we were able to isolate a distinct population of ROSs (58.7%) based on rhodopsin 1D4 (extra-disc), while no staining was observed for 2D4 (intra-disc), indicating no degraded ROSs were present. Regarding COSs (Figure 2g), we were able to isolate an opsin-positive population (8.87%) using anti-opsin PE.

The results of the flow cytometry method were then assessed by analyzing the same batch of POSs on three independent sample preparations (*n* = 3). High repeatability of the method was observed for the analysis of POS purity, with relative standard deviation (RSD) values of 2.2% and 4.7% for ROS and COS populations, respectively.

To demonstrate the purity of the POSs, we generated an in-house batch of retinal extracts from fresh bovine eyes for comparison with the commercial POS batch using flow cytometry. By subjecting the two samples to rigorous testing, we were able to demonstrate the purity of the commercial POS batch compared to the in-house POSs. Using Hoechst and ATPase staining, we demonstrated the presence of nucleated cells and cellular debris in the in-house POSs that were not present in the commercial POSs (Supplementary Figure S2). It is interesting to note that our method can be used to conduct comparative analysis of purity level across different batches of POSs from the same provider while also including batches from different providers.

**Figure 3:**
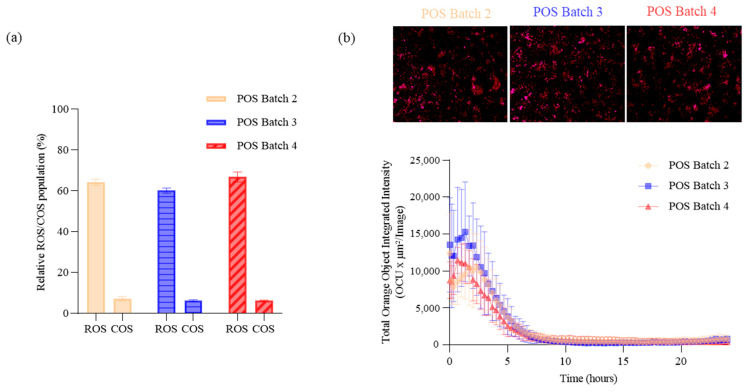
Phagocytosis assessment of different POS batches. (**a**) Relative ROSs and COSs (% of the population) from three different POS batches are represented (batches 2, 3, and 4). Flow cytometry scatter plots representing ROS populations from the three batches are presented in Supplementary Figure S3. (**b**) RPE phagocytosis activity (total integrated intensity) and consistency were monitored for 24 h in the three POS batches using InCucyte scanning.

The POS is a critical raw material for phagocytosis assays, and having a better understanding of batch-to-batch variability will help us better control assay performance. We employed the purity assay to assess the variability of different batches of POSs derived from pools of bovine eyes. This comparison was performed by analyzing three additional POS batches, and each was analyzed on three independent sample preparations (*n* = 3). Data showing the relative ROS and COS populations are shown in Figure 3a. Flow cytometry raw data showing the ROS populations are summarized in Supplementary Figure S3.

The results show similar levels of ROSs and COSs with a relative population of ~60% and ~7%, respectively. Furthermore, the RSD values for each batch in terms of ROS and COS populations are less than 3% and 13%, respectively, again demonstrating the repeatability of the flow-based method. Phagocytosis assay experiments were then performed with these same batches. The data confirm that at equivalent POS purity, the phagocytosis activity of the RPE cells is similar, demonstrating both the reliability of the phagocytosis assay and the flow-based method for monitoring POS purity (Figure 3b).

### 2.3. Study of POS Stability under Several Stress Conditions

**Figure 4:**
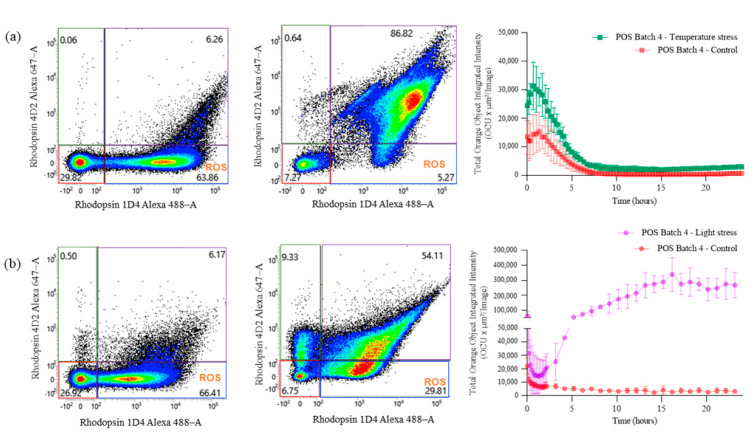
Study of POS stability under heat and light stress conditions. Flow cytometry scatter plots representing POS samples exposed to heat stress at 55 °C for 30 min (**a**) or 6 h of light stress at 750 W/m^2^ (**b**). For each stress condition, controls and stressed POS samples are represented in the left and middle panels, respectively. POSs were differentiated with Rhodopsin 1D4 Alexa 488–A and Rhodopsin 4D2 Alexa 647–A. The panels on the right compare the phagocytosis activity of RPE for 24 h after exposure to the unstressed (blue) or stressed (red) POSs. For the heat stress condition, temperatures of 37, 40, 45, and 55 °C were used, as shown in Supplementary Figure S3. The phagocytosis experiments were performed in triplicate (*n* = 3), and the corresponding error bars based on standard deviation (SD) are reported.

To evaluate whether flow cytometry is a stability-indicating method, POS samples were subjected to forced degradation conditions. Samples were exposed to elevated temperatures or light stress and analyzed via flow cytometry. As shown in Figure 4a, a significant decrease was observed in the ROS population, with a difference of ~60%, after the sample was subjected to heat stress (55 °C, 30 min) compared with the control. The complete data set using different elevated temperatures is presented in Supplementary Figure S4, showing a clear trend of a decreasing ROS population at higher temperatures. Similarly, the light stress sample (750 W/m^2^ for 6 h) also showed a decrease in the ROS population, with a difference of 37%. These data support the use of flow cytometry as a method of indicating POS stability and its use as a purity assay.

To understand the potential impact of POS quality on phagocytosis assay performance, the same stressed POS samples were used for the RPE phagocytosis assay. The results show that the difference in the POS purity directly impacts phagocytosis assay performance. As shown in Figure 4a, the phagocytosis activity of RPE cells along with the degraded POS samples was higher. One hypothesis would be that the disruption of POS material under stressful conditions results in the formation of a large amount of smaller POS debris, providing a larger surface for the pHrodo dye to bind, and therefore, a higher internalization of the dye by RPE cells. Overall, the data demonstrate the necessity to monitor the quality of the POS sample to obtain reliable RPE phagocytosis assay data.

## 3. Discussion

The development of novel cell replacement therapies for retinal diseases, such as hESC- and hiPSC-derived RPE cells, must have morphological and functional properties comparable to those of native RPE cells. Key functional properties of RPE cells include specific markers, polarized secretion of retinal molecules, and phagocytosis activity [4]. Therefore, there is a current demand to develop efficient and reliable analytical assays to characterize these biological functions that would meet regulatory requirements.

To measure RPE phagocytosis activity, several analytical methods, including immunohistochemically staining [15], electron microscopy [19], and flow cytometry [20] can be conducted. However, a time-consuming lysis step involving multiple purification and washing steps is usually required for RPE cells, which makes it difficult to use them in a control system strategy. Here, we report a phagocytosis assay for POSs using pH-sensitive conjugated probes that can directly help to visualize RPE cells via digesting the POS material and thus assess the functional capacity of RPE cells. Different live-cell imaging systems can be employed to measure the RPE phagocytosis activity but, due to its ease to use, the Incucyte^®^ instrument might be more suitable as a quality control assay.

To obtain a reliable and robust RPE phagocytosis assay, it is essential to have a deep understanding of the starting materials used. Therefore, the development of an in vitro phagocytosis assay for POSs requires an analytical method to confirm their identity and effectively measure their purity before assessing RPE cell function.

Extracting POSs from bovine eyes is a crucial step in experimental procedures. However, this process presents inherent challenges due to their sensitivity and fragility, their small size, and their deep retinal location [28]. Studies have reported the delicate nature of POSs, which are prone to damage and disruption during extraction [28]. Temperature changes, light exposure, and mechanical stress have been identified as factors that can affect the structural and functional integrity of POSs [27,29]. In addition, the small size of POSs, which are usually a few micrometers in length, associated with their deep location in the retina poses technical difficulties during isolation. Another critical consideration is the contamination of extracted POSs, which can originate from other retinal layers, adjacent tissues, extra/intracellular proteins, or cellular debris. We demonstrated this crucial step through the in-house retinal extraction method, which we considered a negative control, also confirming the importance of using Hoechst and ATPase antibodies to assess the purity of POS samples (Supplementary Figure S2). These contaminants can interfere with analyses, especially during phagocytosis activity assessment, during which they may be mistaken for engulfed POSs, leading to overestimation or false-positive results for phagocytic events. Furthermore, they can affect the health and viability of RPE cells by inducing cellular stress responses, triggering inflammatory reactions that interfere with normal cellular function. Consequently, the presence of impurities in extracted POS samples can lead to changes in RPE cell behavior, including altered phagocytic activity. This can result in an inaccurate interpretation of the true phagocytic capacity of RPE cells.

Therefore, we decided to develop a flow-based method targeting two specific markers of ROSs and COSs, two main subtypes of POSs. Before analyzing POSs via flow cytometry, one important step is to wash the POS pellets with DPBS to remove all extra/intracellular soluble proteins that could be potentially introduced during the purification process (see Supplementary Figure S5). This additional step reduces the background of the phagocytosis assay since the pHrodo dye used to track the POS pathway can also bind to proteins and other impurities (see Supplementary Figure S1).

The purity of POSs was then assessed through flow cytometry. Using two rhodopsin-specific antibodies, we were able to isolate ROSs by targeting the cytoplasmic *C*-terminal and intra-discal *N*-terminal of the rhodopsin (Figure 1a). We also demonstrated consistency between batches of ROS and COS populations (Figure 3a, Supplementary Figure S3) without any contamination from nucleated cells or other cell debris from the retina.

Heat stress is an important stress to test in biological and pharmaceutical products because it can cause irreversible degradation of the product’s potency and efficacy, but it is still not well documented. Moreover, photoreceptors are specialized cells that are light-sensitive and play a crucial role in visual processing. Given their sensitivity to light, investigating the effects of light on POS integrity is essential to better understand and assess the RPE’s phagocytosis function. Therefore, evaluating the impact of both these stresses during the assay development is important to ensure their quality and efficacy. In Figure 4, we note that upon both heat and light stress conditions, signals for 1D4 and 4D2 improved significantly compared to the control conditions. The increase in 4D2 staining could be explained by the disruption of the POS membrane and higher capacity of the antibody to bind the cytoplasmic *C*-terminal of the rhodopsin, while the increase in 1D4 staining could have resulted from the disruption of free disks and the intra-discal *N*-terminal rhodopsin release. Interestingly, the RPE phagocytosis activity, as determined using degraded POSs, was higher than that of the control sample for both stress conditions. These data suggest that the engulfing mechanism of RPE on POSs is not specific to the intact forms of ROSs and COSs, but can also digest POS fragments. While we observed an important difference in terms of phagocytosis activity (Figure 4), comparing the effects of heat stress and light stress is challenging because the outcomes can vary depending on multiple factors, including the intensity, duration, and specific conditions of each stressor. Generally, light stress is known to have a more pronounced effect on RPE [7]. POS damage induced by exposure to intense light or specific wavelengths can trigger a series of cellular signaling events that stimulate the RPE to upregulate phagocytic receptors and enhance the clearance of damaged POSs [30]. In parallel, heat stress will primarily lead to protein denaturation and disruption of cellular metabolism, and that can explain why we observed a lower phagocytosis activity in this state compared with light stress.

## 4. Materials and Methods

### 4.1. Cell Media, Solvents, and Reagents

Dulbecco’s phosphate-buffered saline (DPBS), FluoroBrite™ Dulbecco’s modified eagle medium with high glucose (DMEM), iMatrix-511 (Nippi), Hanks’ Balanced Salt Solution (HBSS), dimethylsulfoxide (DMSO), HEPES, and high-performance liquid chromatography (HPLC) solvents including water, acetonitrile, isopropyl alcohol, and trifluoroacetic acid (TFA) were all purchased from Thermo Fisher Scientific (Waltham, MA, USA). For flow cytometry, the FACS buffer (DPBS + 0.5% BSA) and bicarbonate buffer (5 mM sodium carbonate, 50 mM sodium bicarbonate) were prepared in-house. The antibodies anti-Rhodopsin (clone 4D2) (Alexa Fluor 647) (CAS No. NBP2-59690AF647), anti-Rhodopsin (clone 1D4) (Alexa Fluor 488) (CAS No. NBP1-47602AF488), and anti-Sodium potassium ATPase alpha 3 (clone XVIF9-G10) (Alexa Fluor 750) (CAS No. NB300-540AF750) were purchased from Novusbio (Centennial, CO, USA); rabbit anti-red/green opsin (clone 1D2) (CAS No. ZRB1265) was purchased from Sigma Aldrich (Darmstadt, Germany), donkey anti-rabbit IgG (PE) (CAS No. 406421) and mouse IgG1 isotype control antibodies (CAS No. MCA1209A647, CAS No. MCA1209A488) were purchased from BioLegend (San Diego, CA, USA). Hoechst 33342 (CAS No. 62249) was purchased from Thermo Fisher Scientific (Waltham, MA, USA). For in-house POS extraction and purification, all the chemicals were purchased from Sigma Aldrich (Darmstadt, Germany) and prepared as described in Papermaster [31].

### 4.2. RPE Cells Preparation

Genentech, Inc. (South San Francisco, CA, USA) supplied the hESC-derived RPE cells used in the study. Each vial contained 2 million RPE cells in a 1 mL cryopreservation medium. Prior to the phagocytosis assay, the frozen fully differentiated RPE cells were thawed and washed in the growth medium (DMEM with 20% Human Serum (HS)). After adjusting the cell concentration, the cell suspensions were mixed with iMatrix-511 (Nippi) and plated in a 96-well black plate (Greiner, Singapore), incubated at 37 °C, 5% CO_2_, for 2–4 days. The growth medium was then replaced with NutriStem^®^ hPSC XF medium (Sartorius, Fremont, CA, USA). The culture medium was changed twice a week. On day 14, confluence and cell morphology were confirmed via microscopy.

### 4.3. Phagocytosis Assay

#### 4.3.1. POS Batches

POS batches were purchased from InVision BioResources (Seattle, WA, USA). The samples were received in 50 mL falcon tubes with aluminum foil covered in a dry-ice box. Each tube contained POSs extracted and purified from 25 bovine eyes. Once arrived, the POS samples were placed on dry ice until thawing. Then, an aliquot was made by taking and transferring 10 µL to each 1.5 mL microcentrifuge tube. The aliquoted POSs were stored at −80 °C.

#### 4.3.2. POS Conjugation with pHrodo™ Dye

For POS conjugation, 10 mM pHrodo™ Red (ThermoFisher Scientific, Waltham, MA, USA) was reconstituted in DMSO following the manufacturer’s standard protocol. On the day of the assay, a POS aliquot was thawed on ice and diluted in 300 µL of DPBS. An amount of 100µL of diluted POS sample (~1 × 10^6^ POS) was then centrifuged at 2000× *g* for 6 min and washed with 0.2 mM sodium bicarbonate buffer, pH 9.2, with gently pipetting several times. After washing, samples were resuspended in pHrodo™ Red working solution (1 µL/sample of ~10 mM pHrodo™ Red added to sodium bicarbonate buffer, pH 9.2) and incubated for 1 h away from light at room temperature. After incubation, the conjugated POS samples were washed three times with HBSS + 0.1% Polysorbate 20 and resuspended in 300 µL/vial of FluoroBrite™ DMEM.

#### 4.3.3. Phagocytosis Assay

One hundred microliters per well of the POS conjugate were added to RPE cells in triplicate. Samples were preincubated for 1 to 2 h at 37 °C with 5% of CO_2_. Wells were washed with 100 µL of EDTA, followed by three washes with PBS. After the last wash, 100 µL/well of DMEM FluoroBrite containing 2 mM HEPES was added. Plates were then placed in the plate holder of an IncuCyte^®^ located in a 37 °C, 5% CO_2_ incubator for IncuCyte scanning or scanned once using the ImageXpress^®^ Micro Confocal (IMX) system. The plates were scanned with the phase contrast and orange channel of the IncuCyte instrument for 24 h. After scanning, the data were analyzed using the IncuCyte software (Sartorius, Gottingen, Germany). The total orange object’s integrated intensity (Y-axis) was generated from each sample as a function of time (X-axis). The plates were scanned around the peak time with the red and green channels of the IMX.

### 4.4. POS Sample Preparation

#### 4.4.1. Heat-Stress-Treated Samples

An aliquot of POS sample was resuspended in 750 µL of DPBS. After gently pipetting to mix, the resuspended POS sample was divided into seven microcentrifuge tubes equally, with 100 µL in each. The tubes were covered with aluminum foil and incubated at different temperatures for 30 min, room temperature, 37 °C, 45 °C, 50 °C, and 55 °C, in a water bath. After incubation, the samples were transferred to wells of a 96-well clear round-bottom microplate and spun down at 2000× *g* for 5 min. The supernatant was collected, and the pellet was stained by antibodies. In parallel, the same process was realized on a second aliquot, except that microcentrifuge tubes were used for the phagocytosis assay after the heat stress treatment.

#### 4.4.2. Light-Stress-Treated Samples

An aliquot of POS sample was resuspended in 300 µL of DPBS. After gently pipetting to mix, the resuspended POS sample was divided into two microcentrifuge tubes equally, with 100 µL in each. Both tubes were exposed to 725 w/m^2^ light generated by the Atlas SUNTEST CPS+ Light Box (Mount Prospect, IL, USA) for 6 h at 23 °C, with the control totally covered with aluminum foil. The samples were transferred to wells of a 96-well clear round-bottom microplate and spun down at 2000× *g* for 5 min. The supernatant was collected, and the pellet was stained with antibodies. In parallel, the same process was performed on a second aliquot, except that after the light stress treatment, both microcentrifuge tubes were used for the phagocytosis assay.

#### 4.4.3. In-House POS Extraction and Purification

Fresh bovine eyes were obtained from Animal Technologies (Tyler, TX, USA). Bovine eyes were received after overnight transport with ice. POSs were then extracted and purified as described by D. S. Papermaster [31]. Briefly, retinas were collected from bovine eyes under dim red light and homogenized. The homogenates were then wrung out and the ROS-containing supernatants were washed and resuspended in 1.10 g/mL sucrose. This 1.10 g/mL sample was added to the top of the gradient consisting of 1.11 g/mL, 1.13 g/mL, and 1.15 g/mL sucrose gradient, followed by ultracentrifugation (106,000× *g* at 4 °C for 30 min). POSs appearing as a single red band at the 1.11/1.13 g/mL interface were collected, washed, and stored at −80 °C pending further use. The POS band and other layers obtained after ultracentrifugation were used as positive control samples for the flow cytometry method.

### 4.5. Characterization of POS Population Using Flow Cytometry

#### 4.5.1. POS Antibody Staining

An aliquot of POS sample was resuspended in DPBS, and a minimum final volume of 100 μL was required. After gently pipetting to mix, 100 µL of resuspended POS sample was transferred to one well of a 96-well clear round-bottom microplate and spun down at 2000× *g* for 5 min. The supernatant was discarded, and the pellet was resuspended in a 200 µL antibody cocktail (1:50 dilution in FACS buffer). For 200 µL, 4 µL of Rhodopsin Antibody (4D2), 4 µL of Rhodopsin Antibody (1D4), 4 µL of Sodium Potassium ATPase Alpha 3 Antibody (XVIF9-G10), 4 µL of Anti-Red/Green Opsin Antibody, and 20 µL of Donkey anti-rabbit IgG Antibody were added and incubated in the dark at 4 °C for 15 min. After incubation, the POS sample was spun down at 2000× *g* for 5 min and resuspended in 200 µL of a new FACS buffer to wash it. The washing step was repeated twice, followed by incubating the sample in 100 µL of Hoechst 33342 solution (1: 10,000 dilution in FACS buffer) in the dark at 4 °C for 15 min.

#### 4.5.2. Flow Cytometry

Experiments were conducted using the FACSLyric™ Flow cytometer (BD Biosciences, San Jose, CA, USA). The first parameters used were the SSC-A (side scatter area) and the FSC-A (forward scatter area), indicating the granularity and size of the cells or particles present in the sample, respectively. The blue laser (488 nm) was used to excite Alexa Fluor^®^ 488 (emission filter 527/32, mirror 507 LP) and PE (emission filter 586/42, mirror 560 LP). The red laser (640 nm) was used to excite Alexa Fluor^®^ 750 (emission filter 783/56, mirror 752 LP) and Alexa Fluor^®^ 647 (emission filter 660/10, mirror 660/10). The Violet laser (405 nm) was used to excite Hoechst 33342, and emission was collected through the filter 448/45, with mirror 448/45. For each sample, a total of 100,000 events were collected. Data were collected and analyzed using BD FACSuite software (BD Biosciences, San Jose, CA, USA). For rhodopsin, gate P3 for impurity was established based on an unstained sample and an isotype control. Gates P2 and P4 were established for disrupted ROSs and ROSs, respectively, based on unstressed samples after staining. For Hoechst 33342 and Na/K ATPase α3, the gates were chosen according to stained cell debris and retinal tissue was obtained from in-house POS extraction and purification. Compensation was processed due to the overlap between the Rhodopsin Antibody (4D2) (Alexa Fluor^®^ 647) and the Sodium Potassium ATPase Alpha 3 Antibody (XVIF9-G10) (Alexa Fluor^®^ 750).

#### 4.5.3. Analysis of Intracellular and Extracellular Soluble Proteins via HPLC/UV

Soluble proteins from POSs resuspended in DPBS were analyzed using HPLC coupled to an ultraviolet (UV) detector. HPLC-UV analyses were performed with Agilent Technologies 1290 Infinity (Santa Clara, CA, USA) using a Waters BioResolve C4 column (150 × 2.1 mm, 2.6 μm) (Milford, MA, USA). Mobile phases were composed of 0.1% (v/v) TFA in water (A) and 70% acetonitrile with 30% isopropyl alcohol (B), with an elution gradient to equilibrate the system as follows: 0 to 2 min, 32%; 2 to 22 min, 32 to 85%; 22 to 24 min, hold at 85%; 24 to 25, 85 to 32%; and 5.5 min. The temperature of the column was set at 70 °C, with a flow rate of 0.4 mL/min. UV detection was performed at 214 and 280 nm using a diode array detector (DAD). The data were analyzed with Agilent MassHunter (B.07.00, Agilent, Santa Clara, CA, USA).

#### 4.5.4. Characterization of POSs Using Microscopy

An aliquot of POS sample was resuspended in 1 mL of DPBS. After gently pipetting to mix, 10 µL of the sample was placed on a glass slide with a coverslip and analyzed immediately using an Olympus bx 61 (Olympus Evident, Tokyo, Japan) with a 100× objective.

## 5. Conclusions

In conclusion, we have shown that our flow cytometry analysis provides a valuable tool for the characterization of photoreceptor outer segments from the bovine retina. We have demonstrated the ability of this method to isolate and identify both rods and cones—two subtypes of POSs—using their specific markers, rhodopsin and opsin proteins, respectively. The use of a negative control such as Hoechst and Na/K ATPase α3 antibodies helped us to exclude cellular debris and other non-POS particles that could result from a bad extraction process and ensured the accuracy and reliability of our analysis. Our results illustrate that flow-cytometry-based methods can provide a detailed and reliable assessment of POS purity and consistency between batches, which has important implications for both basic research and clinical applications. The technology we introduced holds the potential to revolutionize the field of retinal research by offering a robust and efficient way to distinguish between rod and cone outer segments while excluding confounding impurities. Furthermore, the characterization of POSs is an essential component among a set of assays that are necessary for a comprehensive understanding of RPE cell function. For example, our findings suggest that RPE phagocytosis activity—a critical biological function of RPE—could be significantly affected by the purity and integrity of POS samples after heat or light stress. To our knowledge, this is the first demonstration of a flow-cytometry-based method that provides valuable insights into the behavior and interactions of RPE cells with POSs, making it crucial for advancing our knowledge of the role of RPE cells in vision and retinal disease such as AMD. Overall, our study highlights the potential of flow cytometry as a reliable method for the characterization of POSs for RPE phagocytosis assay development and underscores its efficiency for a wide range of industrial and clinical applications in the field of ophthalmology.

## Data Availability

Data available on request due to restrictions eg privacy or ethical. The data presented in this study are available on request from the corresponding author.

## Supplementary Materials

The following supporting information can be downloaded at: https://www.mdpi.com/article/10.3390/ijms241612889/s1.
