# Supplementary material for: Comprehensive Analysis of Photoreceptor Outer Segments: Flow Cytometry Characterization and Stress-Driven Impact on Retinal Pigment Epithelium Phagocytosis
Source: Int J Mol Sci. 2023 Aug 17;24(16):12889. doi: 10.3390/ijms241612889 (PMC10454439; doi:10.3390/ijms241612889)
Supplement: Supplementary file 1 [file ijms-24-12889-s001.zip › ijms-2543349-supplementary.pptx]

## Slide 1
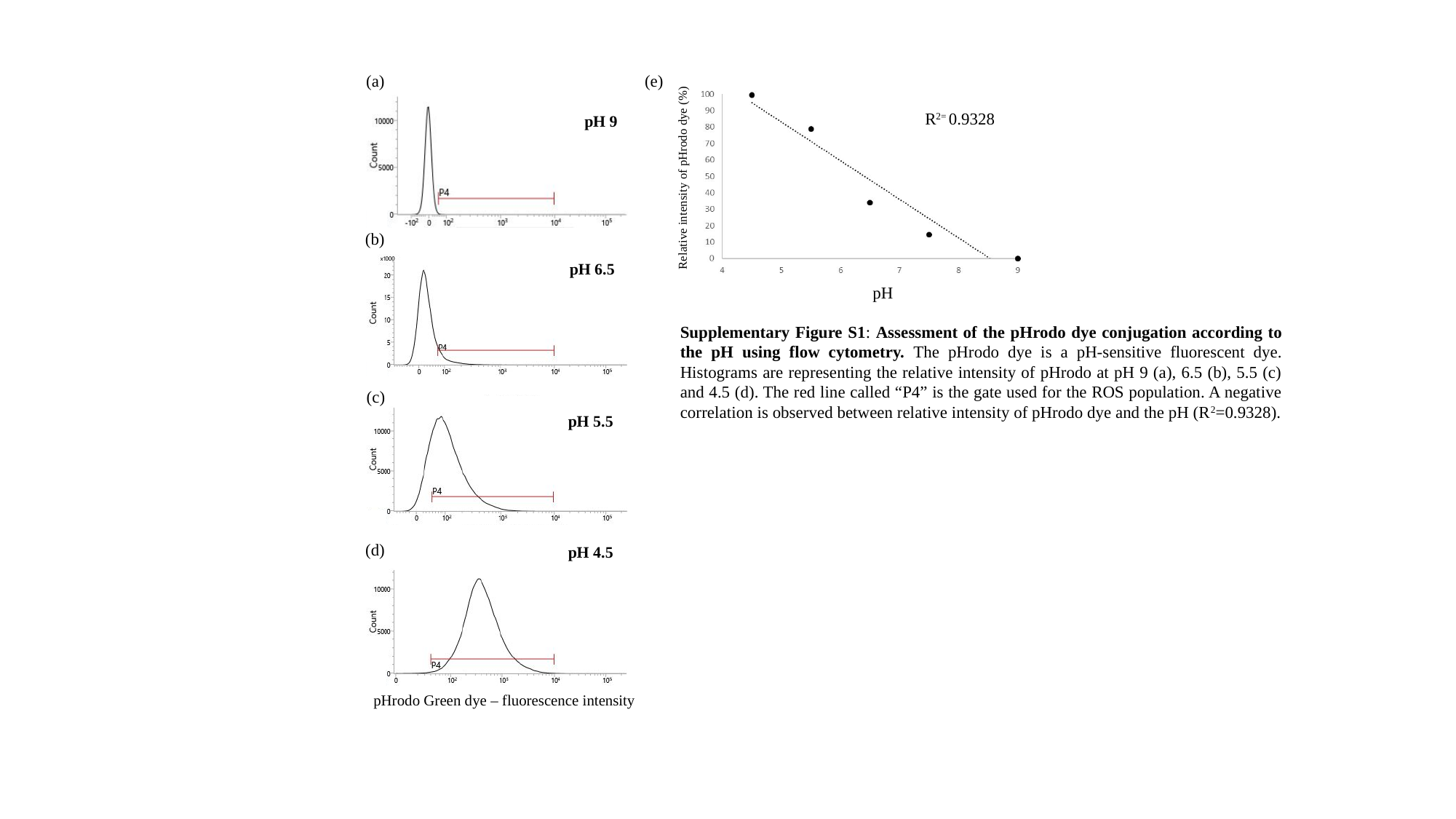

R2= 0.9328
Relative intensity of pHrodo dye (%)
pH
pH 9
pH 6.5
pH 5.5
pH 4.5
(a)
(e)
(b)
Supplementary Figure S1: Assessment of the pHrodo dye conjugation according to the pH using flow cytometry. The pHrodo dye is a pH-sensitive fluorescent dye. Histograms are representing the relative intensity of pHrodo at pH 9 (a), 6.5 (b), 5.5 (c) and 4.5 (d). The red line called “P4” is the gate used for the ROS population. A negative correlation is observed between relative intensity of pHrodo dye and the pH (R2=0.9328).
(c)
(d)
pHrodo Green dye – fluorescence intensity

## Slide 2
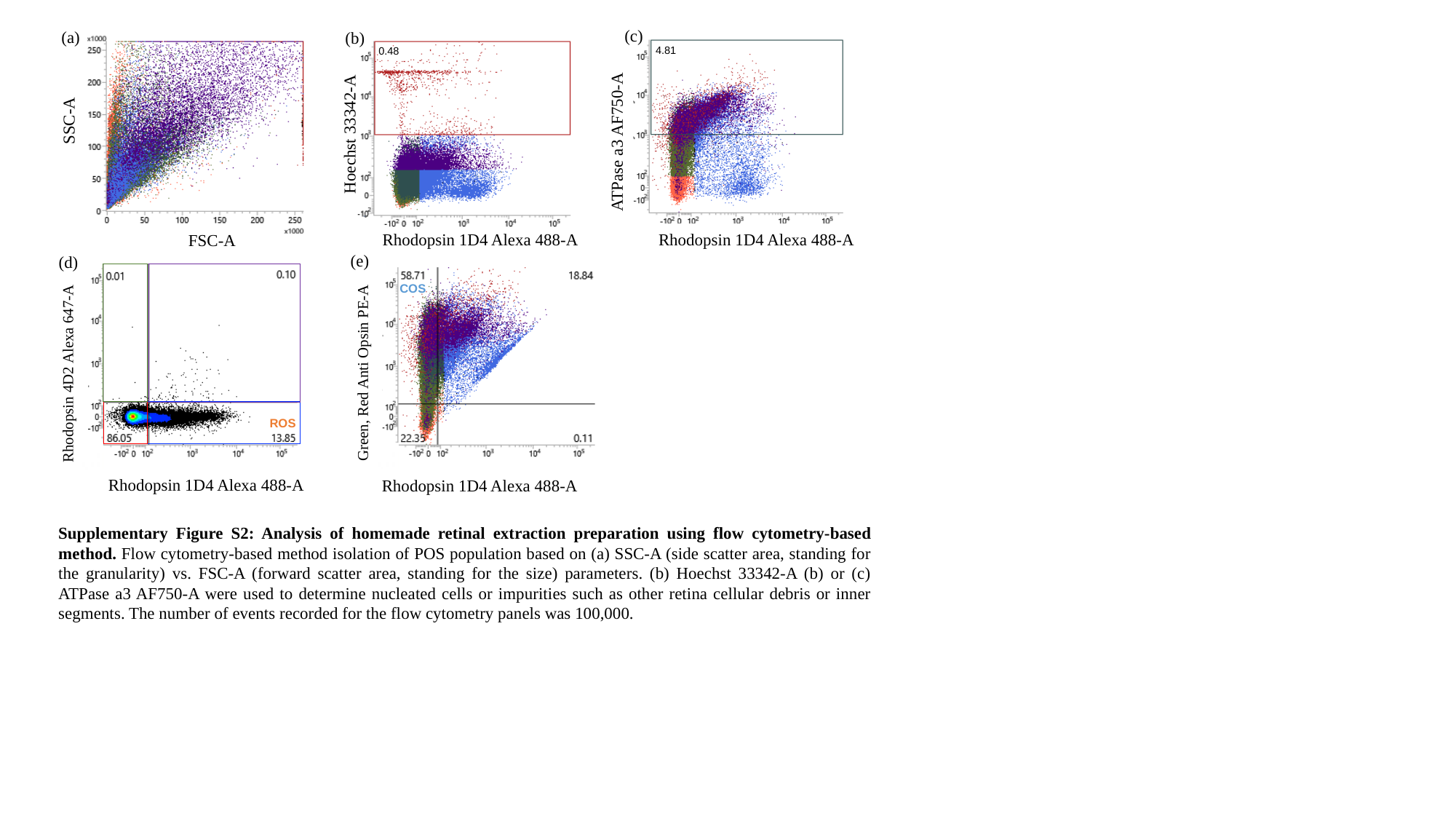

(c)
(a)
(b)
4.81
0.48
SSC-A
Hoechst 33342-A
ATPase a3 AF750-A
Rhodopsin 1D4 Alexa 488-A
Rhodopsin 1D4 Alexa 488-A
FSC-A
(e)
(d)
COS
Rhodopsin 4D2 Alexa 647-A
Green, Red Anti Opsin PE-A
ROS
Rhodopsin 1D4 Alexa 488-A
Rhodopsin 1D4 Alexa 488-A
Supplementary Figure S2: Analysis of homemade retinal extraction preparation using flow cytometry-based method. Flow cytometry-based method isolation of POS population based on (a) SSC-A (side scatter area, standing for the granularity) vs. FSC-A (forward scatter area, standing for the size) parameters. (b) Hoechst 33342-A (b) or (c) ATPase a3 AF750-A were used to determine nucleated cells or impurities such as other retina cellular debris or inner segments. The number of events recorded for the flow cytometry panels was 100,000.

## Slide 3
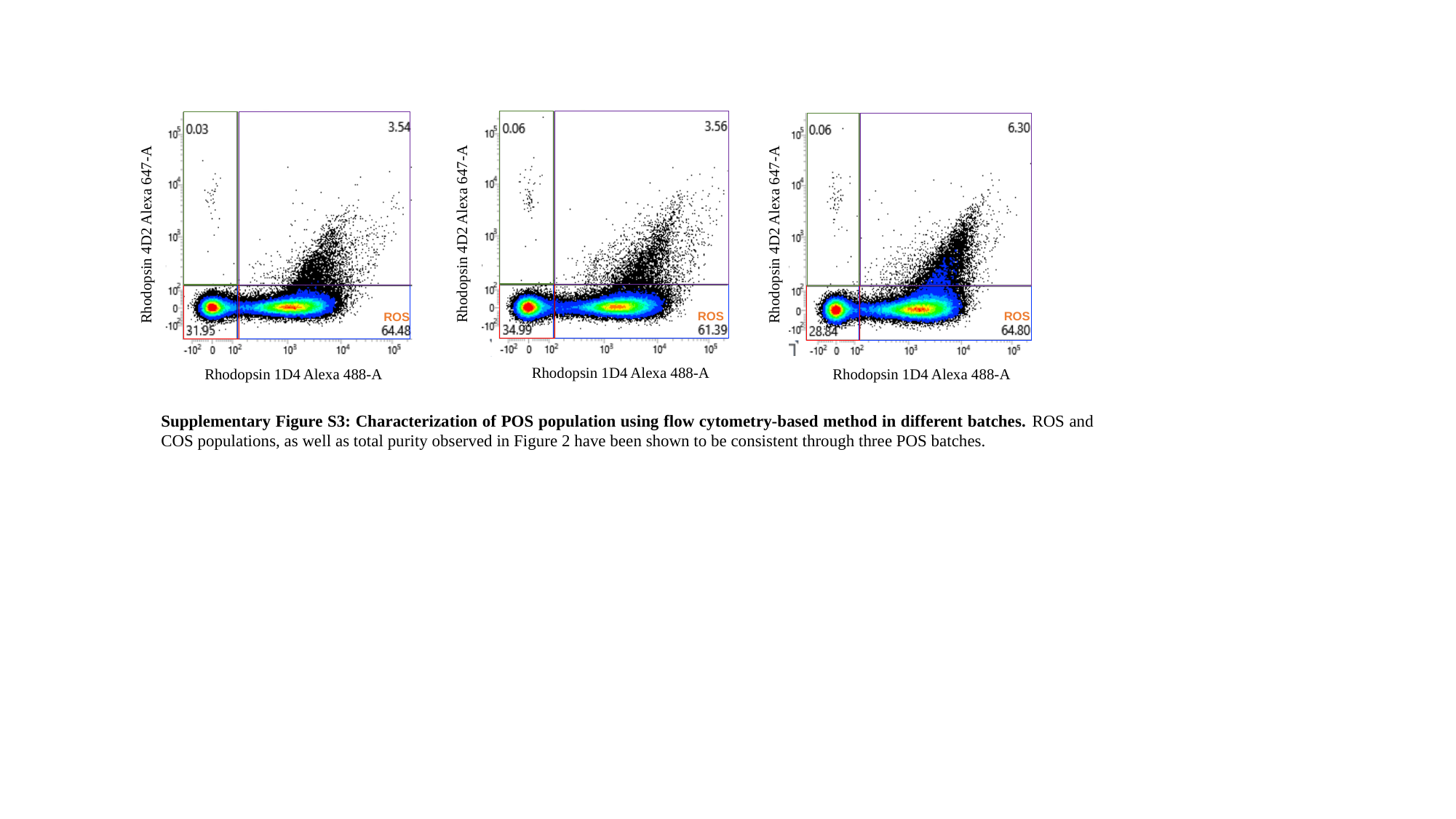

Rhodopsin 4D2 Alexa 647-A
ROS
Rhodopsin 1D4 Alexa 488-A
Rhodopsin 4D2 Alexa 647-A
Rhodopsin 4D2 Alexa 647-A
ROS
ROS
Rhodopsin 1D4 Alexa 488-A
Rhodopsin 1D4 Alexa 488-A
Supplementary Figure S3: Characterization of POS population using flow cytometry-based method in different batches. ROS and COS populations, as well as total purity observed in Figure 2 have been shown to be consistent through three POS batches.

## Slide 4
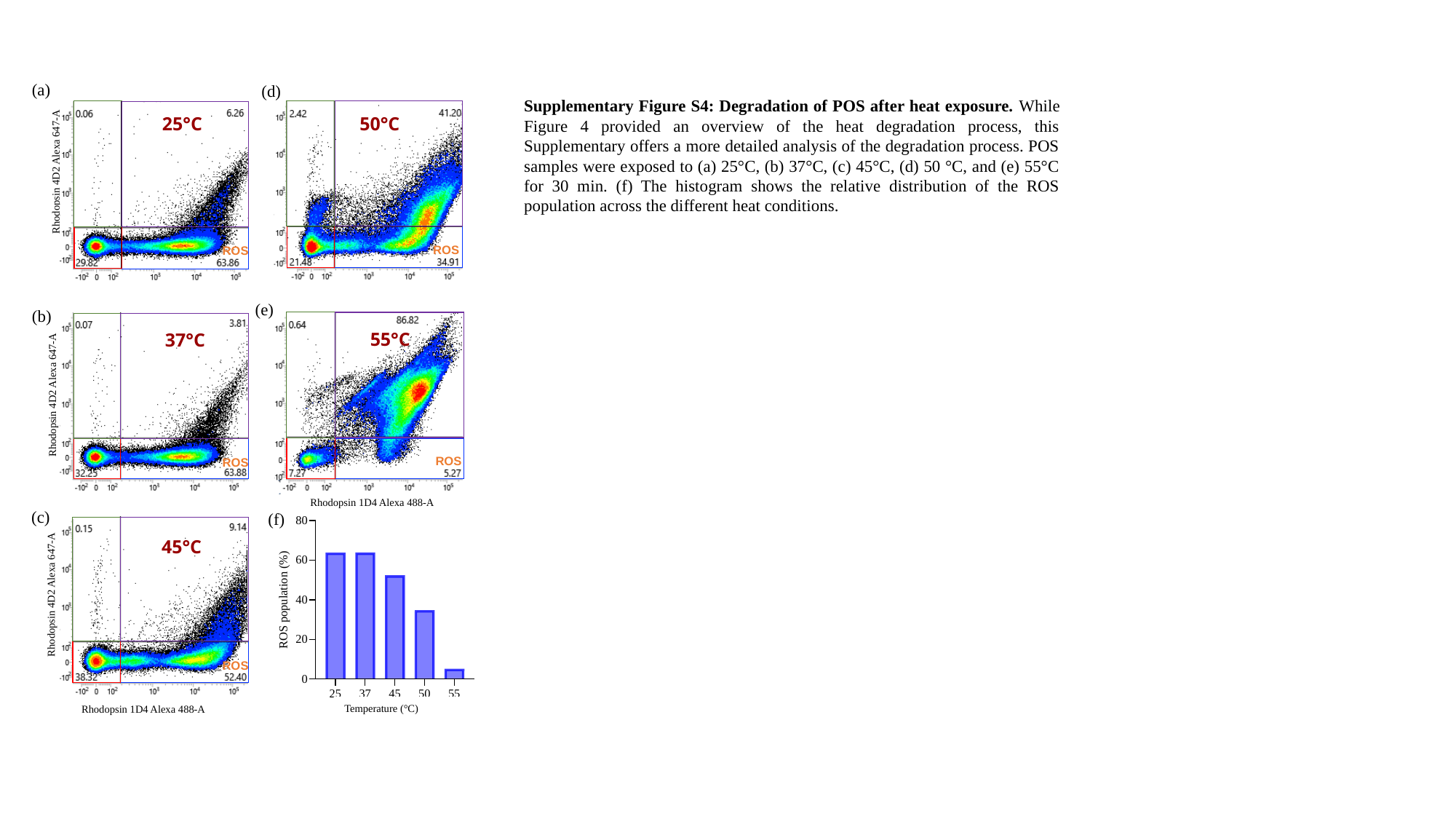

Rhodopsin 4D2 Alexa 647-A
Rhodopsin 4D2 Alexa 647-A
Rhodopsin 4D2 Alexa 647-A
Temperature (°C)
Rhodopsin 1D4 Alexa 488-A
(a)
(d)
(e)
(b)
(c)
(f)
Supplementary Figure S4: Degradation of POS after heat exposure. While Figure 4 provided an overview of the heat degradation process, this Supplementary offers a more detailed analysis of the degradation process. POS samples were exposed to (a) 25°C, (b) 37°C, (c) 45°C, (d) 50 °C, and (e) 55°C for 30 min. (f) The histogram shows the relative distribution of the ROS population across the different heat conditions.
25°C
50°C
ROS
ROS
55°C
37°C
ROS
ROS
Rhodopsin 1D4 Alexa 488-A
45°C
ROS

## Slide 5
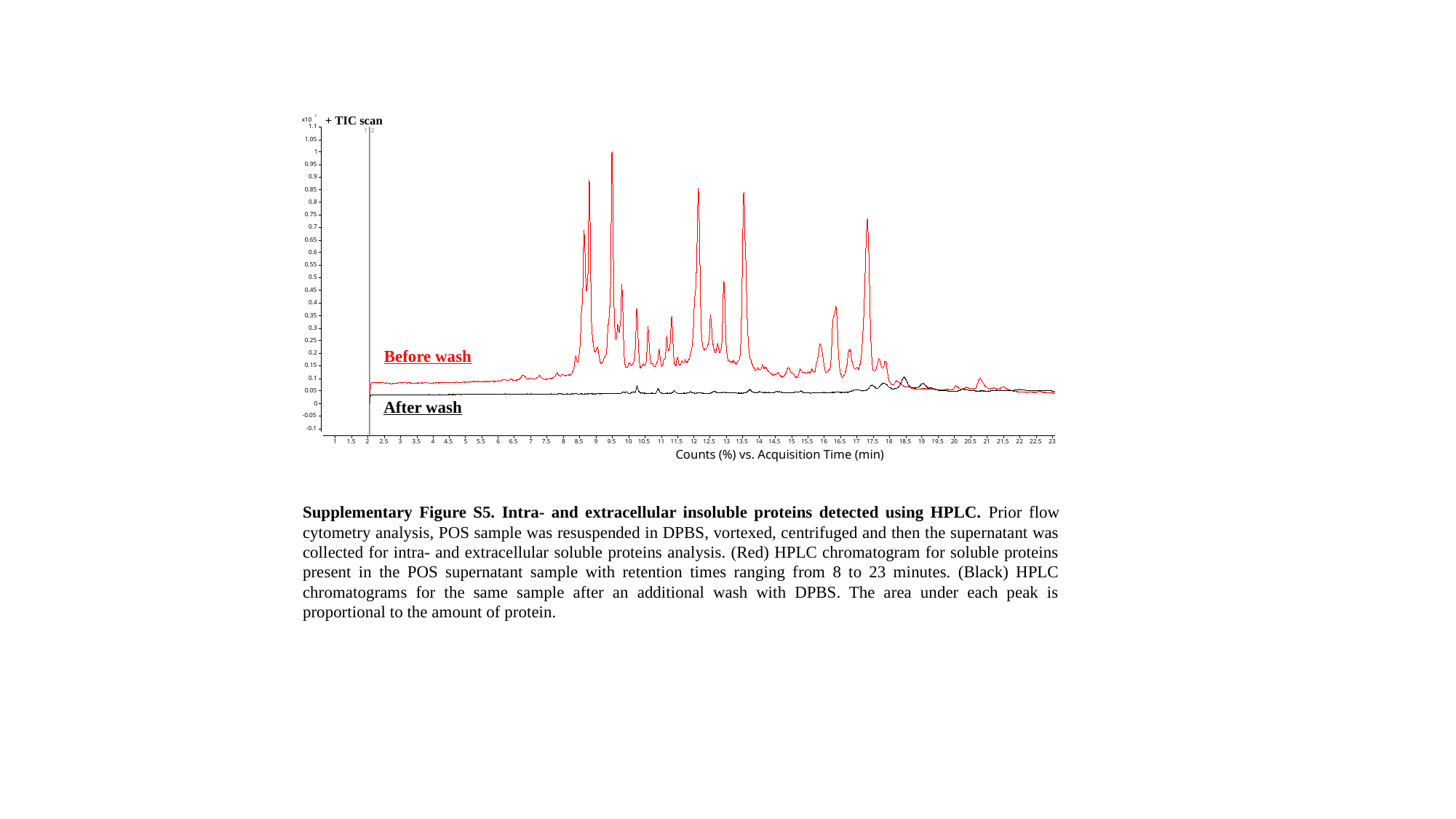

+ TIC scan
2
x10
1.1
1
2
1.05
1
0.95
0.9
0.85
0.8
0.75
0.7
0.65
0.6
0.55
0.5
0.45
0.4
0.35
0.3
0.25
Before wash
0.2
0.15
0.1
0.05
After wash
0
-0.05
-0.1
1
1.5
2
2.5
3
3.5
4
4.5
5
5.5
6
6.5
7
7.5
8
8.5
9
9.5
10
10.5
11
11.5
12
12.5
13
13.5
14
14.5
15
15.5
16
16.5
17
17.5
18
18.5
19
19.5
20
20.5
21
21.5
22
22.5
23
Counts (%) vs. Acquisition Time (min)
Supplementary Figure S5. Intra- and extracellular insoluble proteins detected using HPLC. Prior flow cytometry analysis, POS sample was resuspended in DPBS, vortexed, centrifuged and then the supernatant was collected for intra- and extracellular soluble proteins analysis. (Red) HPLC chromatogram for soluble proteins present in the POS supernatant sample with retention times ranging from 8 to 23 minutes. (Black) HPLC chromatograms for the same sample after an additional wash with DPBS. The area under each peak is proportional to the amount of protein.
